# Comparison of yeast-derived commercial feed additives on *Salmonella* Enteritidis survival and microbiota populations in rooster cecal *in vitro* incubations

**DOI:** 10.1371/journal.pone.0295657

**Published:** 2023-12-14

**Authors:** Margaret Costello, Peter Rubinelli, Jessica Brown, Elena Olson, Dana Dittoe, Si Hong Park, Douglas Korver, Zachary Lawless, Dale Thompson, Steven Ricke

**Affiliations:** 1 Department of Animal and Dairy Sciences, Meat Science and Animal Biologics Discovery Program, University of Wisconsin, Madison, WI, United States of America; 2 Center for Food Safety and Department of Food Science, University of Arkansas, Fayetteville, AR, United States of America; 3 Department of Animal Science, University of Wyoming, Laramie, WY, United States of America; 4 Department of Food Science and Technology, Oregon State University, Corvallis, OR, United States of America; 5 Department of Agricultural, Food, and Nutritional Science, University of Alberta, Edmonton, Canada; 6 Department of Computer Science and Computer Engineering, University of Arkansas, Fayetteville, AR, United States of America; Tokat Gaziosmanpaşa University: Tokat Gaziosmanpasa Universitesi, TURKEY

## Abstract

Yeast-derived products have become more of an interest in the poultry industry as of late because of their use in modulating the gastrointestinal tract (GIT) microbiome to both improve production parameters and prevent infection. This study aimed to evaluate the effects of various yeast-derived products on *Salmonella enterica* inoculation in un *in vitro* rooster cecal incubations and associated effects on the cecal microbiome. Cecal contents were obtained from 53-wk old White Leghorn H & N Nick Chick roosters (n = 3) fed a wheat-based, commercial-type basal diet. Cecal contents were diluted 1:3000 in anaerobic dilution solution (ADS) in an anaerobic chamber, with 20 mL aliquoted to each serum bottle. There were three controls (n = 3): basal diet only, diluted cecal contents only, and basal diet and diluted cecal contents; and five treatments containing the basal diet and diluted cecal contents (n = 3): Citristim® (ADM), ImmunoWall® (ICC), Maxi-Gen Plus® (CBS Bio Platforms), Hilyses® (ICC), and Original XPC® (Diamond V). All treatments were applied at a rate of 2.5 kg/tonne or less. All groups were inoculated with a nalidixic acid-resistant strain of *Salmonella* Enteritidis at 10^7 CFU/mL and incubated at 37 deg C. Samples were collected at 0, 24, and 48 h for *S*. Enteritidis enumeration and 16S rDNA microbial sequencing. *Salmonella* data were log-transformed and analyzed in a two-way ANOVA with means separated using Tukey’s HSD (P≤0.05). Genomic DNA was extracted, and resulting libraries were prepared and sequenced using an Illumina MiSeq. Sequencing data were analyzed in QIIME2 (2021.4) with diversity metrics (alpha and beta), and an analysis of the composition of microbiomes (ANCOM) was performed. Main effects were considered significant at P≤0.05, with pairwise differences considered significant at Q≤0.05. There was an interaction of treatment and time on the enumeration of *Salmonella* where treatments of Citristim, Immunowall, Hilyses, and XPC reduced *Salmonella* by 1 log CFU/mL compared to the controls. At 48 h, each yeast product treatment reduced *Salmonella* by 3 log CFU/mL compared to the controls. There was no main effect of treatment on the alpha diversity metrics, richness, or evenness (P > 0.05). Treatment affected the beta diversity, abundance, and phylogenetic differences, but there were no pairwise differences (P>0.05, Q>0.05). Using ANCOM at the genus level, the taxa *Synergistes*, *Alloprevotella*, *Sutterella*, and *Megasphaera* abundance were significantly different (W = 154,147,145,140, respectively). These results demonstrate the potential of these yeast-derived products to reduce foodborne pathogens, such as *Salmonella* Enteriditis, *in vitro*, without negatively disrupting the cecal microbiome.

## Introduction

Food safety is a central concern in the poultry industry, with pathogens such as *Salmonella* causing outbreaks associated with meat and eggs [1]. Nearly 1 in 25 packages of chicken products at the average grocery store are contaminated with *Salmonella*, and *Salmonella* causes 1.35 million cases of infection every year in the United States (US) [1]. A multi-state S*almonella* Enteritidis outbreak in 2021 connected to not ready-to-eat (RTE) products has refocused producer efforts in preventing foodborne illness [2] again. Because poultry production is almost entirely vertically integrated, focusing on intervention strategies that control *Salmonella* infection from hatching egg production to slaughter reduces the risk of salmonellosis [3].

Roosters are an integral part of breeding systems in commercial flocks. According to breeding recommendations from H & N International, the ratio of roosters to hens is typically between 1:8 and 1:10, with an average hatchability of 80–83%, indicating that H & N roosters are often responsible for upwards of 3,000 chicks [4]. Natural breeding can be cost-friendly and feasible compared to other systems, though it introduces another route of exposure as GIT pathogens such as *Salmonella* can be spread through mating [5]. Sperm pathogens released into the cloaca of a hen can infect the ovaries through the excreta [6], and transovarial transmission of *Salmonella*, specifically *S*. Enteritidis, can lead to infected eggs and eventually more infected birds [7, 8]. Pathogenic bacteria can also affect a rooster’s sperm quality and motility and thus may affect fertility rates in a flock [5]. With higher demands for poultry products, finding methods beyond antibiotics to reduce resistance and improve product quality is critical.

Prebiotics are substrates that provide nutrient sources to gastrointestinal tract (GIT) commensal and beneficial bacterial species [9]. The nutrient sources in prebiotics are thought to provide competitive advantages to bacterial species capable of utilizing them [9]. Pathogen inhibition via prebiotics varies depending on the host microbiome and pathogens present [10]. Gram-positive bacteria, such as lactobacilli, use these nutrient sources and subsequently outcompete Gram-negative bacteria, such as *Salmonella*, *E*. *coli*, and *Campylobacter* [9]. Prebiotics with yeast-derived mannanoligosaccharides function by eliminating movement in *E*. *coli* and *Salmonella* via attachment to the flagella, decreasing binding to the GIT epithelial cells [11]. Prebiotics can also promote short-chain fatty acid (SCFA) synthesis, down-regulating *Salmonella* invasion in GIT epithelial cells [12].

Similarly, various yeast products (byproducts of different fermentation processes such as the production of ethanol for fuel from cereal grains or the production of citric acid) and postbiotics are commercially available for inclusion in poultry diets [13, 14]. Yeast postbiotics are dead or inactivated cells that contain key metabolites that may aid the growth of beneficial bacteria and inhibit *Salmonella* [14]. Other yeast products may offer metabolites without cell debris, and rather than a homogeneous group of products, various preparation processes and whole or fractions of yeast cells have different modes of action. Among the main mechanisms are the binding of pathogens to yeast cell walls [15–17], immunomodulation [18], prebiotic function of compounds such as mannanoligosaccharides [19], provision of nutrients such as nucleotides and nucleosides from yeast cell contents [20], and immune-activating effects of yeast cell wall β-glucans [21].

If effective, yeast-derived products offer an alternative to antibiotics for small and large poultry producers while reducing the risk of foodborne illness. Similar to prebiotics and postbiotics, yeast-derived products also face fewer regulatory barriers as feed ingredients, making them more practical than products classified as drugs. This study tested the effectiveness of various yeast-derived products on reducing *S*. Enteritidis in rooster ceca *in vitro* and to characterize changes to the rooster cecal microbiome.

## Materials and methods

**Fig 1:**
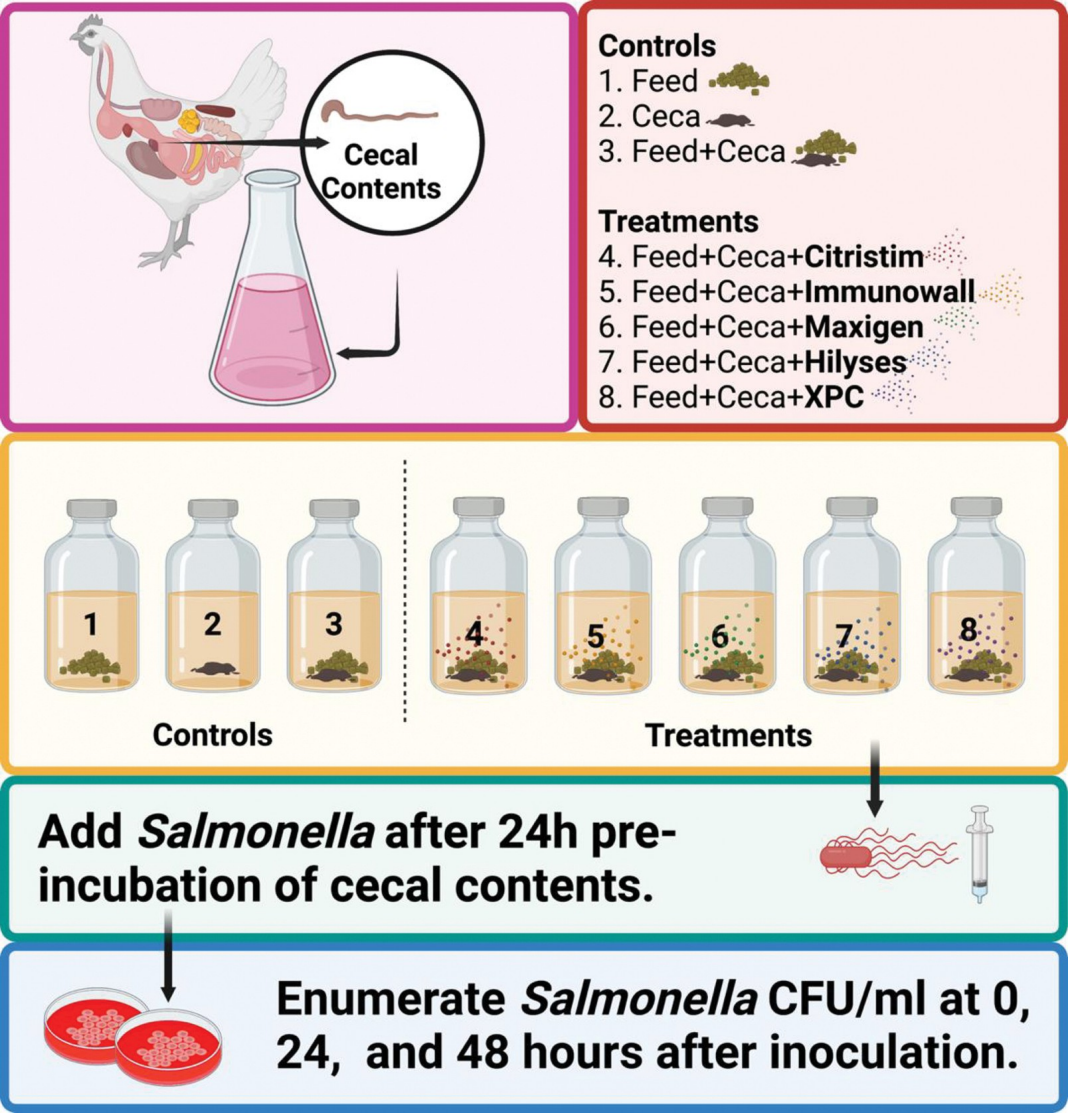
Graphical schematic of the experimental methodologies. Experimental design. Controls contained 1) Feed but no cecal content (“Feed only control”); 2) Cecal content but no feed (“Cecal only control”); 3) no prebiotic but cecal content and feed (“Feed + cecal control”). Experimental units contained feed, cecal content, and different prebiotics as indicated in the Materials and Methods section. Figure created with Biorender.com.

Commercially available yeast-derived products were tested for their ability to inhibit the growth of an intestinal pathogen relevant to human health, specifically the strain *S*. Enteritidis *in vitro*. This was accomplished by using novobiocin and nalidixic acid as selective agents to create an antibiotic-resistant marker strain. A poultry isolate of *S*. Enteritidis type 13A strain was originally obtained from the USDA National Veterinary Services Laboratory (Ames, IA 50011). A spontaneous mutant previously selected [22] to be resistant to nalidixic acid (NA; Sigma, St. Louis, MO) was used in the current study. Different commercial yeast-derived products, representing a range of various active ingredients and modes of action, were screened via an *in vitro* cecal incubation system, as described previously by Rubinelli et al. [23]. A visualization of the methods used for this study is presented in Fig 1.

### Cecal inocula source

A total of 3 Single-Comb White Leghorn roosters (approximately 53 weeks of age; H & N Nick Chick) were housed at the Poultry Research Centre, University of Alberta, and fed the same wheat-based commercial-type laying hen diet as previously described by Korver et al., consisting of 17.0% crude protein, 5.0% fat, and 4.1% fiber with 2,750 kcal ME/kg [24]. This bird study was approved under the experimental procedures outlined in protocol number AUP00000827 by the Animal Care and Use Committee: Livestock of the University of Alberta, following the guidelines set by the Canadian Council of Animal Care in 2009 (Ottawa, ON, Canada). Ceca (including contents) were collected using aseptic technique into individual sterile Whirl-Pak bags, frozen at -20°C, and shipped on dry ice to the University of Arkansas, Fayetteville, AR, for analysis. Simultaneously, a sample of the experimental diets described previously by Korver et al. (2023) was shipped to the University of Arkansas [24]. The ceca were thawed and subsequently stored at 4°C until transferred to an anaerobic chamber (Coy Laboratory Products, Grass Lake, MI, USA).

### Media preparation

The mixed cultures from the rooster ceca were grown in Anaerobic Dilution Solution (ADS) consisting of 0.45g/L K_2_HPO_4_, 0.45 g/L KH_2_PO_4_, 0.45 g/L (NH_4_)_2_SO_4_, 0.9 g/L NaCl, 0.1875 g/L MgSO_4_-7H_2_O, 0.12 g/L CaCl_2_-2H2O, 1 mL/L 0.1% resazurin, 0.05% cysteine-HCl, and 0.4% CO_2_-saturated sodium carbonate, with the sodium carbonate added last. The ADS was subsequently equilibrated with an anaerobic gas mixture (80% nitrogen, 10% carbon dioxide, 10% hydrogen) for 30 minutes in an anaerobic chamber using an aquarium air pump and air stone, and autoclaved at 121 121°C, 15 psi, for 20 minutes. The ADS was then left to cool to room temperature before equilibrating in an anaerobic chamber devoid of oxygen, containing an atmosphere of 87% nitrogen, 10% carbon dioxide, and 3% hydrogen. Two palladium catalyst scrubbers running continuously maintained an anaerobic environment inside the chamber.

### Test products

The control treatment and the five yeast-derived products tested as various treatments. The treatments were a commercial-type, wheat-based laying hen ration fed to the roosters housed at the Poultry Research Centre, University of Alberta (FC); cecal contents only, the basal diet and cecal contents, and various treatments with both the basal diet and cecal contents: Immunowall at 0.5 kg/tonne (yeast-derived product derived from *Sacchromyces cerevisiae* containing 20% mannooligosaccharides and 35% β-glucans; ICC, São Paulo, Brazil); Hilyses at 2.5 kg/tonne (hydrolyzed yeast-derived product from *S*. *cerevisiae* containing free amino acids, nucleotides, peptides, mannanoligosaccharides, and β 1,3-glucans, ICC, São Paulo, Brazil); Citristim at 1.0 kg/tonne (a *Pichia guilliermondii* postbiotic that is a coproduct of citric acid production, containing whole yeast, nucleic acids, mannans, and β-glucans, ADM, Decatur, IL); the control diet with Maxi-Gen Plus at 1.0 kg/tonne (a processed yeast product with β 1,3-glucans and mannan carbohydrates; CBS BioPlatforms Inc., Calgary, AB, Canada); and the control diet with Original XPC at 2.5 kg/tonne (a postbiotic derived from *S*. *cerevisiae* fermentation products with various metabolites including peptides, proteins, antioxidants, phytoserols, and nucleotides; Diamond V, Cedar Rapids, IA). Each control and treatment were tested on three individual ceca from the three roosters.

### Dilution and preparation of cecal contents

The five yeast-derived feed additives were each added to separate 20-mL aliquots of ADS + 1:3000 diluted cecal contents as described below to a final concentration of 1% (w/v), and the control ration was added to each 20 mL of ADS + cecal contents to a final concentration of 1.25% (w/v).

A portion of the cecal contents was removed aseptically within the chamber, weighed, and diluted 1:3000 by adding 0.1 grams of cecal content to 300 mL ADS. After, 20 mL of the diluted cecal content was transferred to each of the 21 serum bottles with feed and yeast products as indicated above. Three additional cultures received sterile ADS and feed without cecal contents or yeast-derived to serve as controls.

### *Salmonella* Enteritidis inoculation

An initial inoculum of approximately 1 x 10^7^ CFU/mL of a nalidixic acid–resistant (NAR) marker strain, SE13A, of *S*. Enteritidis was added to each 20 mL culture. Cultures were removed from the anaerobic chamber and placed in a shaking incubator at 37°C at 200 rpm for 48 hours using airtight rubber stoppers and aluminum crimps.

The cecal bacteria isolated from the University of Alberta roosters were pre-incubated for 24 hours with feed and their respective treatments, and *S*. Enteritidis was added after 24 hours. Three control cultures for each cecum tested were run in parallel. At 0, 24, and 48 hours, an aliquot of each culture was removed, diluted, and spread on Brilliant Green Agar medium (BD Biosciences) supplemented with 20 μg/mL nalidixic acid for quantification of colony forming units (CFU) of the marker strain, *S*. Enteritidis 13A per mL of the culture (Fig 1). Before adding the marker strain of *S*. Enteritidis, the diluted cecal contents were also tested for nalidixic acid-resistant bacteria via direct plating. No nalidixic acid-resistant bacteria were detected. Cultures were plated at each time point to determine the presence of *S*. Enteritidis 13A. If it was not found, the cultures were inoculated into TT enrichment broth to further confirm the absence of *S*. Enteritidis 13A.

### Microbiome 16S Sequencing

After polymerase chain reaction (PCR) analyses and confirmation on a 1.5% agarose gel, a library was prepared using the V4 region of the 16S rRNA gene, as detailed in Kozich et al. [25]. Normalization was conducted on the PCR products using a SequalPrep™ Normalization kit (Life Technologies, Carlsbad, CA, USA). Subsequently, 5 μL of each DNA sample was added to a pooled plate library, and the concentrations were found by the KAPA Library Quantification Kit (Kapa Biosystems, Woburn, MA, USA). The pool was simultaneously evaluated using an Agilent 2100 Bioanalyzer System (Agilent, Santa Clara, CA, USA). Using 20 pM HTI buffer and 0.2N fresh NaOH, 6 pM of a final concentration of the diluted library and PhiX Control v3 (Illumina, San Diego, CA, USA) was generated. The diluted sample was added to the PhiX control v3 (5%, v/v), and 600 μl of the solution was added to the MiSeq v2 (500 cycles) reagent cartridge (Illumina, Carlsbad, CA, USA).

### Microbiota bioinformatic analysis

Data sequences were uploaded onto the BaseSpace Website (Illumina, San Diego, CA, United States), where sequence run quality and run completion was determined. Di-multiplexed data was downloaded locally and uploaded onto QIIME2-2021.4 via the Casava1.8 paired-end pipeline [26]. Data were visualized and trimmed in DADA2 using the chimera consensus pipeline. Alpha and beta diversity were computed via the QIIME phylogeny align-to-tree-mafft-fasttree methodology and then analyzed for all available metrics of alpha and beta diversity via QIIME diversity core-metrics-phylogenetic with a sampling depth of 6150 that was able to retain 35% features in 96% of the samples. The sampling depth was confirmed with alpha rarefaction plots. Taxonomic assignment of the operational taxonomic units was conducted using classify-sklearn provided by QIIME2-2021.4 SILVA database with a confidence limit of 95% [27, 28]. Alpha diversity was analyzed for richness with the Shannon Diversity Index and evenness via Pielou’s Evenness [29, 30]. The alpha diversity analytics included the Kruskal-Wallis tests for pairwise differences within the variables and analysis of variance (ANOVA) to test for the interactions between variables [31]. The beta diversity metrics were assessed with quantitative indicators, such as Bray-Curtis dissimilarity index and Weighted Unifrac distance matrix [32], using the Analysis of Similarity (ANOSIM) function, which considers the mean variation of the population and dispersion [33]. Significant features were plotted along the X-axis and visualized using the Emperor PCoA plots. The interaction for beta diversity metrics was analyzed using permutational multivariate analysis of variance (ADONIS). The differential abundance was identified via ANCOM analysis [34]. Microbiota main effects were considered significant if the main effect had P < 0.05 and the pairwise effect had Q < 0.05 with each statistical measurement within the QIIME2-2021.4 pipeline. The Q-value represented the P-value adjusted for a strict false discovery rate and was incorporated into the QIIME2-2021.4 pipeline. The feature table, rooted tree phylogenetic, and taxonomy were brought into R Studio (R Studio 2023.03.1+446; R 4.3.1). A heat map (ggplot2) was produced, and core microbiome analyses were completed (phyloseq, microbiome utilities), with core members identified at a detection setting of 0.01 with a prevalence of more than 50% [35]. Alpha diversity and taxonomy figures were generated in Microsoft Excel (Microsoft, Redmond, WA, USA), while the remaining figures were created using other software: methods figure (Biorender), PCOA plots (QIIME2), heatmap (R Studio), and Venn Diagrams [36].

## Results and discussion

### Effect of yeast products on reduction of *Salmonella*

Roosters are kept on commercial layer breeder farms to produce fertile eggs, although they offer another route of exposure to GIT pathogens that can be transmitted via insemination [7]. The rooster reproductive tract can be infected by its own GIT pathogens, particularly *Salmonella* since the infected excreta reaches the cloaca [6, 8]. Rooster sperm has naturally occurring commensal and pathogenic microorganisms that can impact semen motility and quality and potentially infect hens through reproduction [5]. *S*. Enteritidis, present in semen, has the potential to colonize hens’ ovaries, allowing for vertical transmission through contaminated yolks [8, 37]. *S*. Enteritidis more commonly attaches itself to the yolk membrane, and as the fertilized egg incubates, the yolk membrane loses integrity, and a gradual flow of nutrients along with *S*. Enteritidis migrate into the embryo [8, 37]. Vertical transmission of *S*. Enteritidis causes economic cost and foodborne illness concerns through lowered fertility, less productive hens, and eventually infected poultry products [37, 38].

Pathogenic bacteria from the GIT, such as *Campylobacter*, *Clostridium perfringens*, and *Salmonella*, are common and often naturally occurring in poultry semen [39]. *Salmonella* can attach to the spermatozoa on the midpiece or the tail, though usually not the head due to the high amounts of oligosaccharides that have been shown to inhibit *Salmonella* binding to gut epithelial cells [40, 41]. Chicken sperm have longer tails than their mammalian counterparts to aid in motility from the cloaca to the sperm storage tubules [42]. Because of this, *Salmonella* decreases sperm motility and viability, negatively impacting fertility [40]. Since *Salmonella* is a GIT pathogen, dietary interventions offer the most promising solution. This study tests yeast-derived products and their effectiveness against *S*. Enteritidis *in vitro* to reduce *Salmonella* in the rooster GIT. Because of this potential transmission relationship, employing cecal contents in an *in vitro* system to screen the effects of different treatments on mitigating an *S*. Enteritidis in the presence of rooster cecal microbiota is a practical initial step to determine if feed additives such as yeast-based products would sufficiently restrict proliferation of *S*. Enteritidis beyond the GIT.

To best represent poultry husbandry in Western Canada, a wheat-based diet was fed to the roosters prior to cecal extraction. Wheat-based diets are frequently utilized in western Canada due to their availability and prospective health benefits [43]. High-fiber diets have been shown to reduce ammonia emissions when compared to soybean and corn diets, enhance energy utilization, improve amino acid utilization and nutrient digestibility, and lower feed costs depending on the region [44–47]. Commensal GIT microbiota benefit from high-fiber diets through fermentation products such as SCFAs and some vitamins, including vitamin B complex and K [48]. In a wheat bran study, *Lactobacillus* and *Bifidobacterium* were positively affected, which may enhance SCFA synthesis, and wheat brans interacted well with xylo-oligosaccharide prebiotics, demonstrating the viability of feed additives on non-corn-based diets [49]. The current study used a wheat-based diet to reflect the commercially popular diet in Western Canada. While this study did not compare the effects of the yeast-derived products between different basal diets, understanding how the basal diet interacts with yeast-derived products should be investigated for poultry producers to know how to optimize the effects of feed additives.

Yeast-derived products are not only beneficial to the GIT microbiome but also to the host immune system. For instance, Citristim is associated with healthy immune responses in turkeys as under normal, healthy conditions, the prebiotic encourages immune tolerance and promotes the immune system during necessary inflammatory responses [18]. A limitation of testing these products *in vitro* was the absence of host immune response data, which would have offered more insight into the apparent *Salmonella* reductions and microbial taxonomy as a function of the host response.

**Fig 2:**
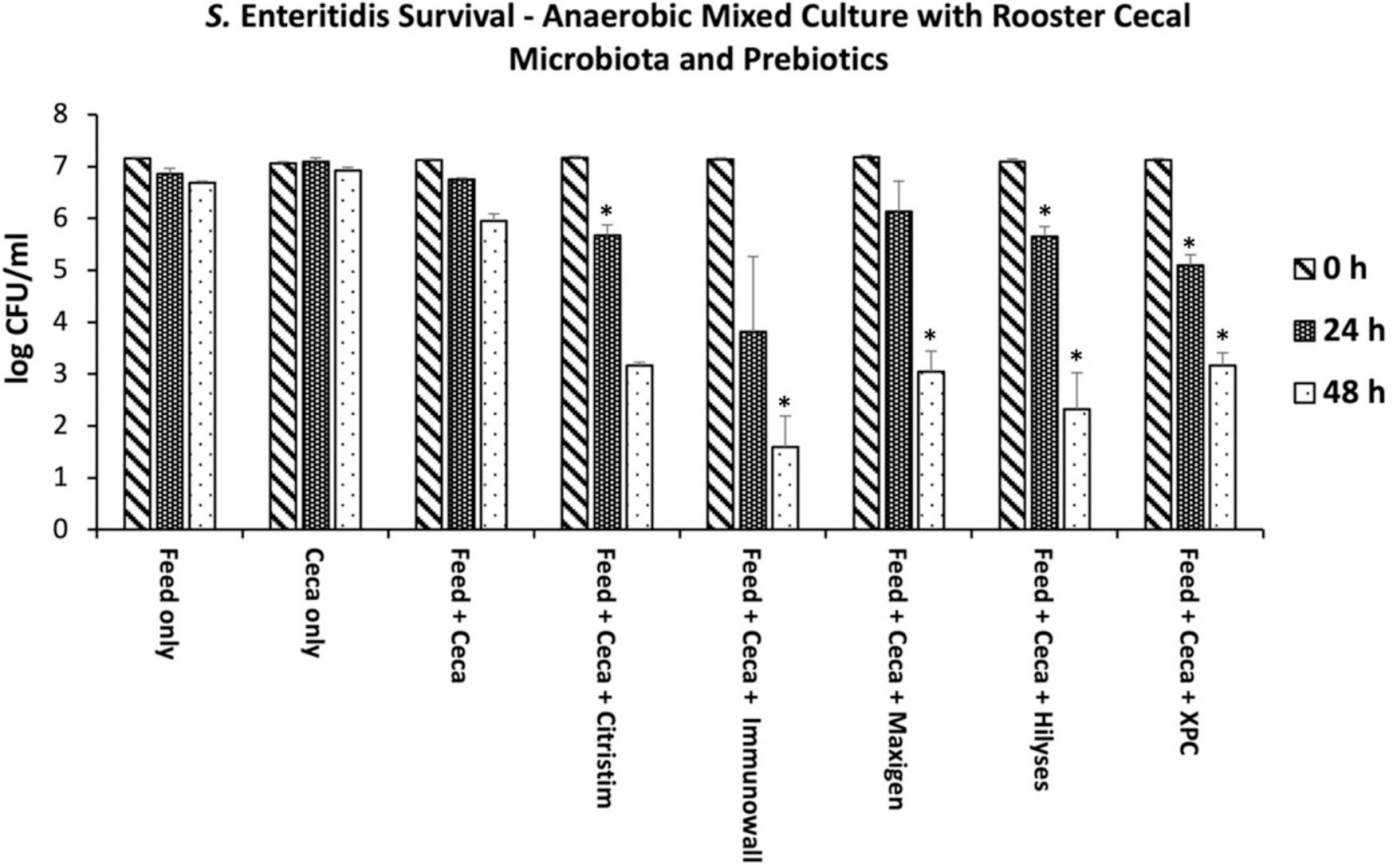
*S*. Enteritidis enumeration at 0h, 24h, and 48h. Survival of *Salmonella* Enteritidis strain SE 13A in *in vitro* mixed anaerobic cultures with and without prebiotics. The treatments included a commercial-type, wheat-based laying hen ration fed to the roosters housed at the Poultry Research Centre, rooster cecal contents only, feed and cecal contents only, the control diet with Citristim at 1.0 kg/tonne, the control diet with Immunowall at 0.5 kg/tonne, the control diet with Maxi-Gen at 1.0 kg/tonne, the control diet with Hilyses at 2.5 kg/tonne, and the control diet with XPC at 2.5 kg/tonne. Data points and brackets represent three biological replicates’ mean and standard error. At 48 hours, Hilyses and Immunowall had one and two ceca, respectively, with no detectable *Salmonella* by direct plating and TT enrichment but were recorded as “1 CFU/ml” because zero could not be graphed on a logarithmic scale. In some cases, lower error bars appear longer than upper error bars due to plotting on a log scale. Asterisks are included that show significance between the treatments and the feed and cecal contents only control.

When the GIT tract bacteria metabolize yeast-derived products, the resulting fermentations have been shown to inhibit *Salmonella* [23]. Postbiotics are inactivated or dead cells that may include metabolite products that benefit the host, and molecules present in postbiotics can both directly and indirectly impact the local microbiota with their antimicrobial properties, effects on quorum sensing, and availability to improve GIT barrier function, in the case of SCFAs [14]. In the current study, each of the five yeast-derived products, when introduced to the rooster cecal microbiota, inhibited *S*. Enteritidis SE13A (Fig 2). Citristim, Hilyses, MaxiGen, and XPC were inhibited to a comparable extent by 48 hours post-*Salmonella* inoculation: approximately three logarithms lower than the control treatment with no product added. At 48 hours post-inoculation, Immunowall appeared to be somewhat more effective, with an additional log reduction compared to the other products. At 48 hours, Hilyses and Immunowall had one and two ceca, respectively, with no detectable *Salmonella*. This was confirmed by tetrathionate enrichment.

Interestingly, this study used lower inclusion rates than typical cecal *in vitro* studies to be more representative of industry standards while also operating in the scope of an *in vitro* design. For instance, Diamond V recommends that their product, XPC, be fed at 0.125% of the diet, with lower inclusion rates as the bird matures, and ADM Animal Nutrition recommended their product, Citristim, be fed at inclusion rates from 0.05%-0.20% [50, 51]. While the inclusion rates in this study ranged from 0.05%-0.25%, slightly higher than commercial recommendations, other studies testing various prebiotics and postbiotics *in vitro* had inclusion rates as high as 7.5% [23, 52]. The inclusion rates for the present study emphasize that inhibition of *S*. Enteritidis still occurs even at lower inclusion rates.

These results indicate that some fermentation of the yeast-derived products by the cecal microbiota is required to maximize the inhibitory impact of the respective products. This is unsurprising, as we have observed this with previous yeast product studies using broiler cecal inocula. Examining the prebiotic-like yeast fermentation product XPC in broiler cecal studies, Rubinelli et al. (2016) observed increases in certain SCFAs that correlated with decreased survival of *Salmonella* under those conditions suggesting that the yeast product influences fermentation patterns, and depending on the SCFA, can be more or less inhibitory to *Salmonella*. SCFAs are known to be antagonistic to *Salmonella* and other pathogens in the GIT when applied as antimicrobials [53–56]. Prebiotics, such as mannooligosaccharides, can also inhibit pathogenic bacteria such as *Salmonella* because of direct binding to lectins on the type 1 fimbria, obstructing movement [57]. Given the similarities in inhibition among the five yeast products used in the current study, it would be interesting to compare the respective SCFA patterns generated in each fermentation versus the control to determine if each of the four yeast products resulted in similar SCFA profiles after fermentation.

### Effect of yeast products on rooster cecal microbiota

In recent years, considerable progress has been made in developing and using next-generation sequencing technology to understand microbial communities better. A significant advancement has been using 16S rDNA microbiome sequencing to identify individual members of microbial communities in a wide range of ecosystems and environments. This has been particularly impactful on food production systems, including the GIT microbial populations in food animals [58]. As more bioinformatics techniques have been developed, the opportunities to use these approaches to examine feed additives’ impacts and other production parameters on the poultry GIT have advanced considerably [59]. This becomes particularly useful as an analytical tool for differentiating poultry cecal responses to feed additives with potentially similar mechanisms. While overall *S*. Enteritidis inhibition appeared similar among the four yeast-derived products, it is conceivable that different yeast products stimulate different members of the cecal microbiota even if they possess similar functions and fermentation properties.

**Fig 3:**
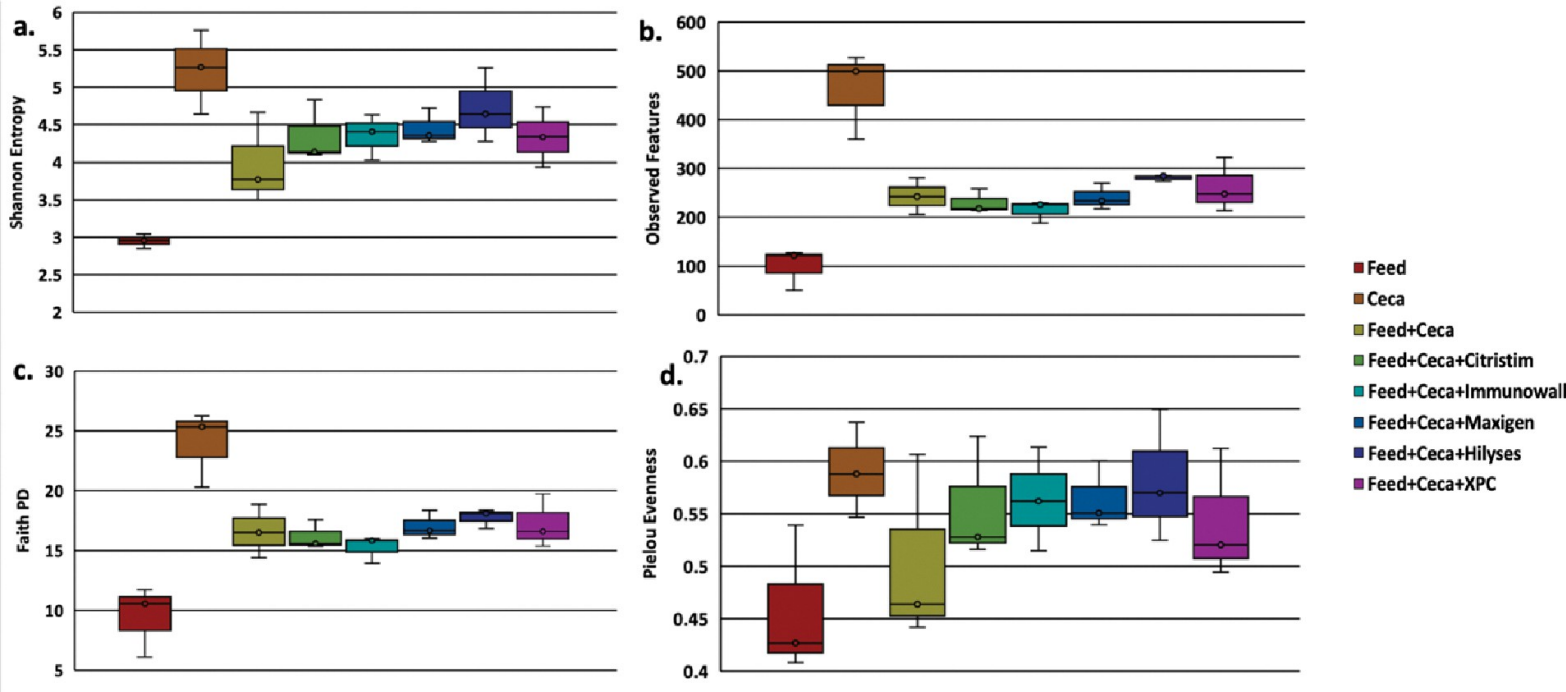
Alpha diversity of the *in vitro* cecal cultures. A comparison of alpha diversity between treatment groups. Shannon’s entropy (a), observed features (b), Faith’s phylogenetic diversity (c), and Pielou’s evenness (d) shown using ANCOM analysis with significance at (P < 0.05). Treatments include feed alone; cecal contents alone; feed and cecal contents without treatment, and feed and cecal contents with various treatments: Citristim, Immunowall, Maxigen, Hilyses, and XPC. There was no alpha diversity significance between the feed and cecal contents treatment and any of the treatments.

**Fig 4:**
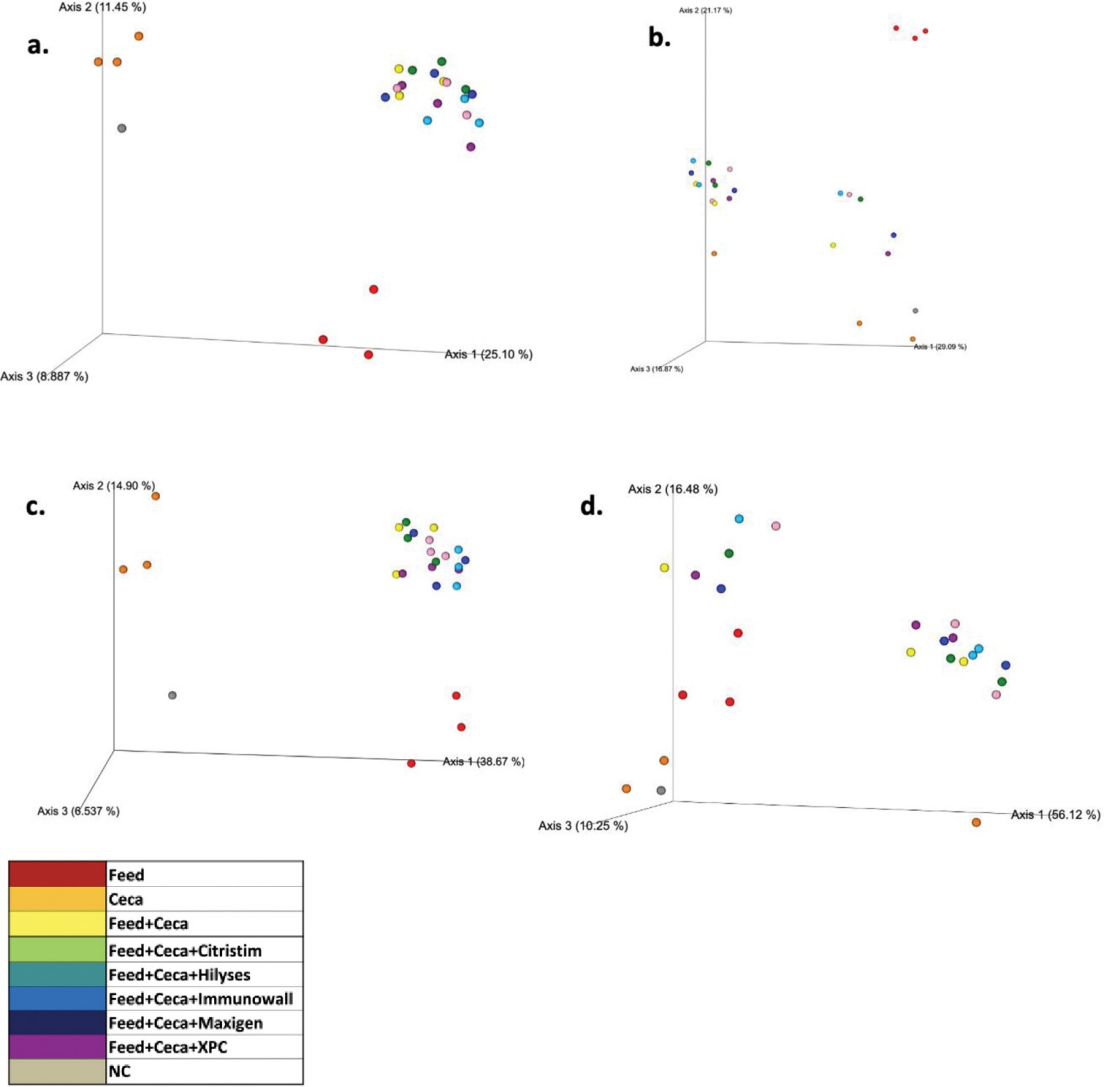
The main effect of beta diversity on Jaccard distance, Bray-Curtis dissimilarity, unweighted unifrac, and weighted unifrac. A comparison of beta diversity metrics using ANOSIM between treatment groups. Jaccard (a), Bray-Curtis (b), unweighted unifrac (c), and weighted unifrac (d) were all measured, and significance was determined at (P < 0.05). Different colors were used to represent the treatment groups: red represents feed, orange represents ceca, yellow represents feed+ceca, green represents with feed+ceca+Citristim, teal represents feed+ceca+ Hilyses, light blue represents feed+ceca+Immunowall, dark blue represents with feed+ceca+Maxigen, purple corresponds with feed+ceca+XPC, and gray represents the negative control. There was no beta diversity signficance.

Microbiome 16S rDNA sequencing was conducted on the rooster *in vitro* cecal incubations to determine the level of diversity response within each yeast product incubation and comparisons among the different yeast products. Based on Faith PD alpha diversity metrics, there was a significant effect of the yeast product on cecal microbiota (P < 0.05, Fig 3), and there was a trend for significance based on Shannon’s Entropy (P = 0.075, Fig 3). This would indicate that each yeast product was equally supportive of a relatively diverse cecal microbial population. However, no significant pairwise differences were detected among the yeast product types based on the same alpha metrics (Q > 0.05, Fig 3). ANOSIM results for the beta diversity metrics indicated significant abundance and phylogenetic differences between the samples (Bray-Curtis, P = 0.001, Fig 4; Weighted Unifrac, P = 0.005, Fig 4). It would appear that each yeast product supported somewhat distinct microbial populations when compared with each other.

**Fig 5:**
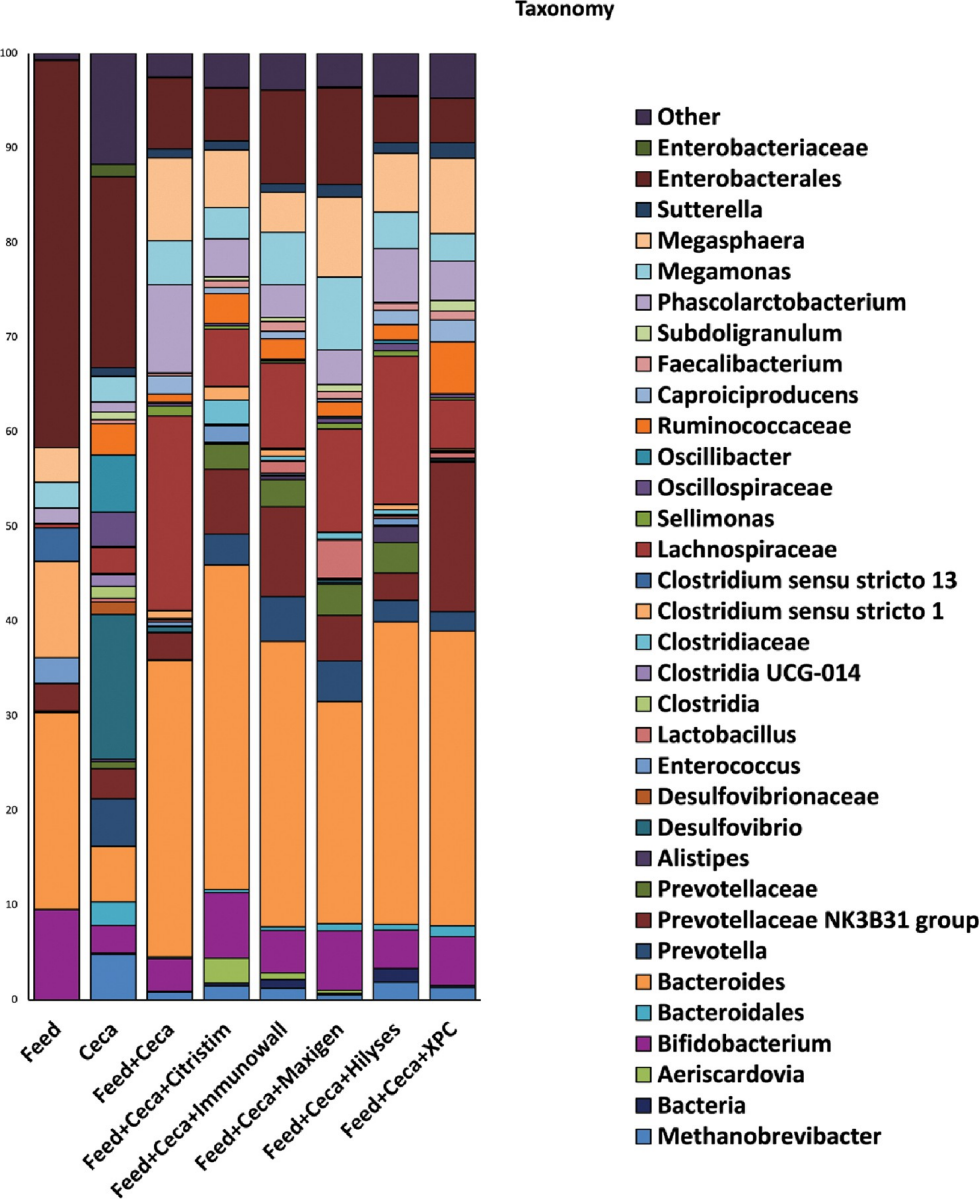
Taxonomic relative abundance of the genera of the treatment groups. The ANCOM analysis on the genera level representing the median relative abundance within the different treatment groups. Treatments include feed only, cecal contents only, feed and cecal contents only, and several treatments with feed and cecal contents: Citristim, Immunowall, Maxigen, Hilyses, and XPC.

Several distinct taxa were identified when individual bacteria were identified from the bioinformatics analyses (Fig 5 Taxa Bar plots). *Enterobacterales* was abundant in the feed with no yeast-derived product added group, and while it was present in the other treatment groups, it was of the highest abundance in the feed. *Bacteriodes* was also present in the treatment groups, especially in the groups treated with yeast products. *Lachnospiraceae* was also detected in all treatment groups, along with *Phascolarctobacterium* and *Megasphaera*. Based on ANCOM analysis for differential abundance on the genera level, there were four significantly different taxa in relative abundance compared to the 186 identified taxa associated with the treatment groups: *Alloprevotella*, *Megasphaera*, *Suterella*, and *Synergistes*. *Alloprevotella* were abundant only in the ceca and drastically decreased in all other treatments (Fig 5). *Megasphaera* were more prevalent in the FC treatment group and relatively reduced in all other treatments except for the XPC group. Both *Lachnospiraceae* and *Megasphaera* are commonly found in chicken cecal populations. *Lachnospiraceae* are carbohydrate utilizers and produce SCFA and, therefore, could be considered antagonistic to *Salmonella* [53]. A butyrate producing *Megasphaera* spp. has also been identified in chicken ceca, and *M*. *elsdenii* has been examined as a potential probiotic to control *Salmonella* in ruminants [60, 61]. These results complement denaturing gradient gel electrophoresis (DGGE) findings from an *in vivo* laying hen study with band numbers identifying similarly identified taxonomy: *Bacillus*, Firmicutes, *Lactobacillus*, and *Clostridium* [24]. Other *in vitro* and *in vivo* studies yielded similar findings. Citristim has been shown to increase *Lactobacillus* populations in cecal cultures [18]. A cecal *in vitro* experiment testing XPC also found *Lachnospiraceae* bacteria, while also reporting higher relative abundances of *Ruminococcus*, *Oscillospira*, *and Enterobacteriaceae* [62]. XPC has also been tested *in vivo* for its effects on the cecal microbiome, where elevated abundances of *Lachnospiraceae* and *Enterobacteriaceae* were observed [63]. Park et al. also observed *B*. *fragilis*, a *Bacteriodes* species noted for producing succinic and acetic acids, known for aiding gastrointestinal health [63]. Further examination of these specific groups of cecal microorganisms and whether they can mechanistically respond to specific yeast-derived products used in the study needs to be determined.

### Core microbiome

**Fig 6:**
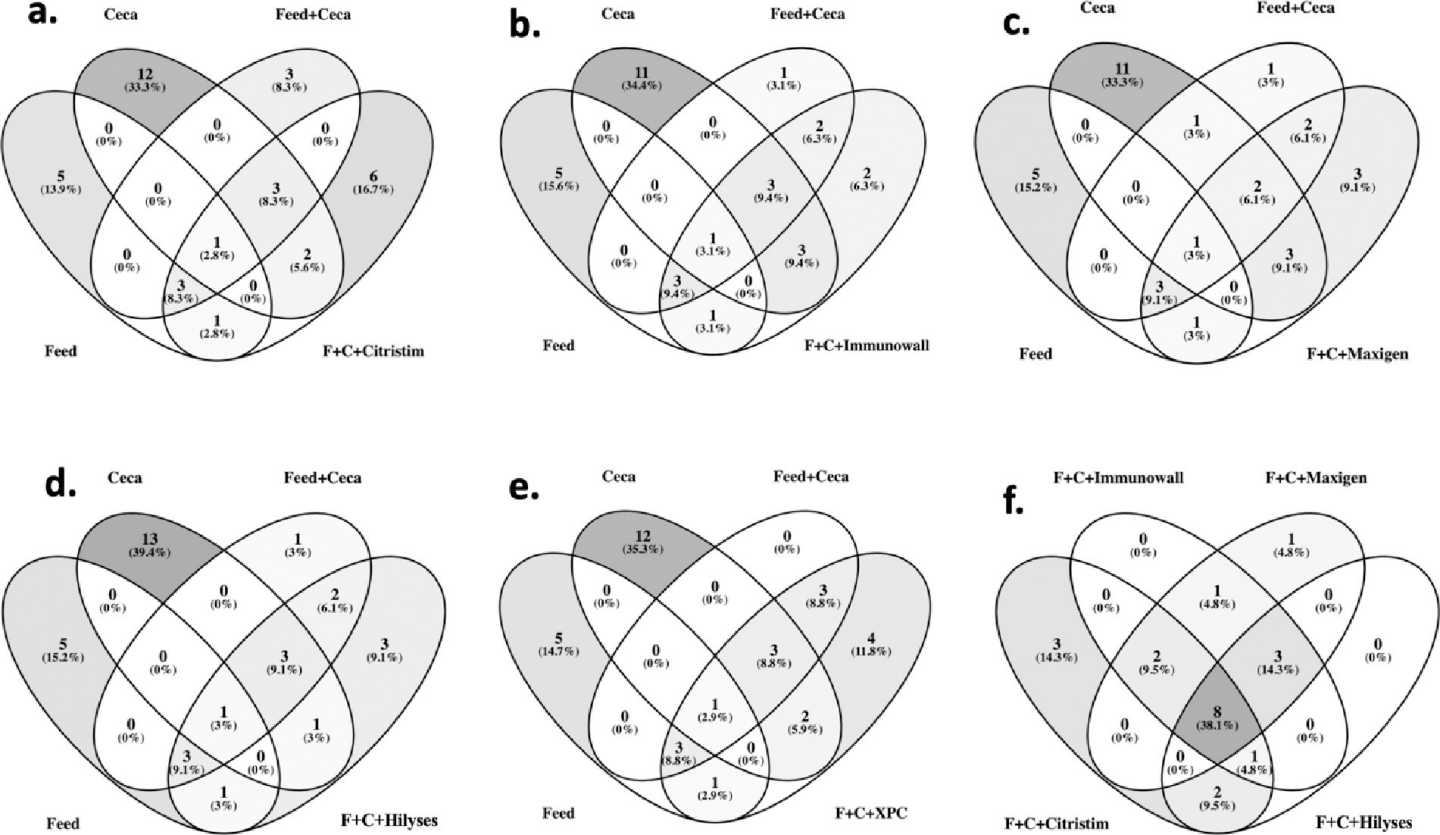
Comparison of shared core microbiome members. Venn diagrams demonstrating the core microbiome commonalities between treatment groups and controls: **a)** Citristim compared to the controls, **b)** Immunowall, **c)** Maxigen, **d)** Hilyses, **e)** XPC, and **f)** the four prebiotic treatments, Citristim, Immunowall, Maxigen, and Hilyses. Parameters were set at a detection rate of 1% with prevalence in at least 50% of all samples in a treatment.

Analyses were run with a detection of 0.01 and prevalence at 50% of the samples to determine core ASVs (Table 1) to determine the core microbiome members. These results are shown in a series of Venn diagrams displaying the similarities between the treatment groups and controls (Fig 6). The five treatment groups shared ten ASVs belonging to six families: *Bacteroidaceae*, *Acidaminococcaceae*, *Selenomonadaceae*, *Veillonellaceae*, *Lachnospirales*, and *Prevotellaeceae*, and they shared these ASVs with the FC control. Compared to the controls, Citristim produced the most unique ASVs with five unique microorganisms, including *Prevotellaceae*, *Sutterella*, *Enterococcus*, *Clostridium*, and *Lachnospiracheae*. Both Maxigen and Immunowall had identical ASVs identified in the Ruminococcaceae family. Ruminococcaceae can break down and degrade various polysaccharides and fibers, generating SCFAs available for intestinal epithelial cells and preventing pathogenic growth through a lowered pH [64]. Several other core members present in all treatment groups are beneficial to both growth parameters and pathogen control. One genus conserved across all groups was *Bacteriodes*, which is positively associated with the intestinal IgA response in broilers by enhancing the expression of several genes involved in the IgA response [65]. Specifically, *B*. *fragilis* has been identified as beneficial to gastrointestinal health through succinic and acetic acid production and has been considered in human health as a probiotic to combat intestinal inflammation [63, 64]. Veillonellaceae and Prevotella have both been associated with diets containing gluten, concurring with the core microbiome results of this study, which used a wheat diet as opposed to corn [65, 66]. Removing gluten short-term from the human diet has been correlated with a decreased abundance of Veillonellaceae, and when cattle are fed fermented corn gluten-wheat diets, there is an increase in Prevotella and Veillonellaceae abundances [65, 66]. Both families are known propionate producers, contributing to gut integrity and health [66–68].

**Table 1:** Core microbiome of both control and treated ceca present in 50% of samples in a treatment as shown as amplicon sequence variants (ASVs)^1^.

Treatment	Organisms	ASV
**Feed**	*Bacteriodes*	a4ad693cd3dadd5f680a73e2fec1e867
** **	*Bacteriodes*	5599f7ca5067653572b626c8aa23a364
** **	*Bacteriodes*	840a763739f0fad66d7f93a222512069
** **	Enterobacterales	942c3786f7fac261cd1b917a7f8ae302
** **	Enterobacterales	70d67fe656d527767789833f8187ebf8
** **	Enterobacterales	0e7abc22c6649b647f11fab3b27b4fbf
** **	*Enterococcus*	f0016306c5b5c843d7a744ce57de1fe5
** **	*Megasphaera*	37d28c8bef2c4a48da6f73a6cbbb9458
** **	*Clostriduim_sensu_stricto_1*	734109cf9adf9652dd01e52810ac68b8
** **	*Clostriduim_sensu_stricto_1*	839533e4f50bd02833e041872066facc
**Ceca**	*Methanobrevibacter*	8188f1a29237f8225edc05ebc003d694
** **	*Synergistes*	1e0a2a190ddf01123d79d4c4730d31c3
** **	*Bifidobacterium*	ad94483d0ef754adc0de920cc3da376e
** **	Bacteriodales	a7884d4b2624d82707c1977586e4edda
** **	*Prevotella*	1ca777a8f73852eb9ba10dc81df7629b
** **	*Prevotellaceae_NK3B31*	afb2d76ff3f015fd627cfbe2c569e255
** **	*Alloprevotella*	39209d925d7c9119a99bd54d453bab9f
** **	*Bacteriodes*	840a763739f0fad66d7f93a222512069
** **	*Desulfovibrionaceae*	5956385a9908b8d2c24e04c6c32a0cf8
** **	*Desulfovibrio*	f8402cf86f88626fc936bb589d30ee89
** **	*Desulfovibrio*	c0a6c473de379fed7b19b2803e8643a0
** **	Enterobacterales	59cc1a95b2eb626e91ef3252671a244d
** **	*Enterobacteriaceae*	464b00cad6510559cdc6f3ed7474d89a
** **	*Phascolarctobacterium*	84928c196f82f7a75135b6b70cd08cb9
** **	*Megamonas*	2272d56d77c26103fc92d40454184a3c
** **	*Oscillibacter*	7aae0931a0cfe3517339678d4c148c62
** **	*Oscillibacter*	2376d19002347dfa4c4dd6dd4da30c6f
** **	Oscillospiracaea	07b1d37c1c3d685f6a8d3aa3df63e787
**Feed+Ceca**	*Prevotellaceae_NK3B31*	afb2d76ff3f015fd627cfbe2c569e255
** **	*Bacteriodes*	a4ad693cd3dadd5f680a73e2fec1e867
** **	*Bacteriodes*	840a763739f0fad66d7f93a222512069
** **	Enterobacterales	0e7abc22c6649b647f11fab3b27b4fbf
** **	*Phascolarctobacterium*	84928c196f82f7a75135b6b70cd08cb9
** **	*Megamonas*	2272d56d77c26103fc92d40454184a3c
** **	*Megasphaera*	37d28c8bef2c4a48da6f73a6cbbb9458
** **	*Caproiciproducens*	558ce6d49f66d4103395bc6499188464
** **	*Lachnospiraceae*	3929267c8fe1994d4402da434c0cfa63
** **	*Lachnospiraceae*	ae90a23b25d7af4ddfbf59e38aa59d61
**Feed+Ceca+Citristim**	*Methanobrevibacter*	8188f1a29237f8225edc05ebc003d694
** **	*Bifidobacterium*	ad94483d0ef754adc0de920cc3da376e
** **	*Prevotellaceae_NK3B31*	afb2d76ff3f015fd627cfbe2c569e255
** **	*Prevotellaceae*	a76983a3bf38708183f845241cce869d
** **	*Bacteriodes*	a4ad693cd3dadd5f680a73e2fec1e867
** **	*Bacteriodes*	5599f7ca5067653572b626c8aa23a364
** **	*Bacteriodes*	840a763739f0fad66d7f93a222512069
** **	*Sutteralla*	9c79fdab4df936067fa14dd300da7ab8
** **	Enterobacterales	0e7abc22c6649b647f11fab3b27b4fbf
** **	*Enterococcus*	ddd8f8c09a18fa7364d8685cbb426a3d
** **	*Phascolarctobacterium*	84928c196f82f7a75135b6b70cd08cb9
** **	*Megamonas*	2272d56d77c26103fc92d40454184a3c
** **	*Megasphaera*	37d28c8bef2c4a48da6f73a6cbbb9458
** **	*Clostridium_sensu_stricto_1*	d3689cc3386028ff63a489d99bf8b855
** **	*Lachnospiraceae*	137aed16f7b5d28b9d31f26c6ea9682a
** **	*Lachnospiraceae*	3929267c8fe1994d4402da434c0cf
**Feed+Ceca+Immunowall**	*Methanobrevibacter*	8188f1a29237f8225edc05ebc003d694
** **	*Bifidobacterium*	ad94483d0ef754adc0de920cc3da376e
** **	*Prevotella*	1ca777a8f73852eb9ba10dc81df7629b
** **	*Prevotellaceae_NK3B31*	afb2d76ff3f015fd627cfbe2c569e255
** **	*Prevotellaceae*	a76983a3bf38708183f845241cce869d
** **	*Bacteriodes*	a4ad693cd3dadd5f680a73e2fec1e867
** **	*Bacteriodes*	5599f7ca5067653572b626c8aa23a364
** **	*Bacteriodes*	840a763739f0fad66d7f93a222512069
** **	Enterobacterales	0e7abc22c6649b647f11fab3b27b4fbf
** **	*Phascolarctobacterium*	84928c196f82f7a75135b6b70cd08cb9
** **	*Megamonas*	2272d56d77c26103fc92d40454184a3c
** **	*Megaspaera*	37d28c8bef2c4a48da6f73a6cbbb9458
** **	Ruminococcaceae	4812652729e476ac697097d8ebb954b1
** **	*Lachnospiraceae*	3929267c8fe1994d4402da434c0cfa63
** **	*Lachnospiraceae*	ae90a23b25d7af4ddfbf59e38aa59d61
**Feed+Ceca+Maxigen**	*Methanobrevibacter*	8188f1a29237f8225edc05ebc003d694
** **	*Bifidobacterium*	ad94483d0ef754adc0de920cc3da376e
** **	*Prevotella*	1ca777a8f73852eb9ba10dc81df7629b
** **	*Prevotellaceae_NK3B31*	afb2d76ff3f015fd627cfbe2c569e25
** **	*Prevotellaceae*	a76983a3bf38708183f845241cce869d
** **	*Bacteriodes*	a4ad693cd3dadd5f680a73e2fec1e867
** **	*Bacteriodes*	5599f7ca5067653572b626c8aa23a364
** **	*Bacteriodes*	840a763739f0fad66d7f93a222512069
** **	Enterobacterales	0e7abc22c6649b647f11fab3b27b4fbf
** **	*Phascolarctobacterium*	84928c196f82f7a75135b6b70cd08cb9
** **	*Megamonas*	2272d56d77c26103fc92d40454184a3c
** **	*Megasphaera*	37d28c8bef2c4a48da6f73a6cbbb9458
*** ***	Ruminococcaceae	4812652729e476ac697097d8ebb954b1
*** ***	*Lachnospiraceae*	3929267c8fe1994d4402da434c0cfa63
*** ***	*Lachnospiraceae*	ae90a23b25d7af4ddfbf59e38aa59d61
**Feed+Ceca+Hilyses**	*Prevotella*	1ca777a8f73852eb9ba10dc81df7629b
** **	*Prevotellaceae_NK3B31*	afb2d76ff3f015fd627cfbe2c569e255
** **	*Prevotellaceae*	a76983a3bf38708183f845241cce869d
** **	*Bacteriodes*	a4ad693cd3dadd5f680a73e2fec1e867
** **	*Bacteriodes*	5599f7ca5067653572b626c8aa23a364
** **	*Bacteriodes*	840a763739f0fad66d7f93a222512069
** **	*Sutteralla*	9c79fdab4df936067fa14dd300da7ab8
** **	Enterobacterales	0e7abc22c6649b647f11fab3b27b4fbf
** **	*Phascolarctobacterium*	84928c196f82f7a75135b6b70cd08cb9
** **	*Megamonas*	2272d56d77c26103fc92d40454184a3c
** **	*Megasphaera*	37d28c8bef2c4a48da6f73a6cbbb9458
*** ***	*Lachnospiraceae*	137aed16f7b5d28b9d31f26c6ea9682a
*** ***	*Lachnospiraceae*	3929267c8fe1994d4402da434c0cfa63
*** ***	*Lachnospiraceae*	ae90a23b25d7af4ddfbf59e38aa59d61
**Feed+Ceca+XPC**	*Methanobrevibacter*	8188f1a29237f8225edc05ebc003d694
** **	*Bifidobacterium*	ad94483d0ef754adc0de920cc3da376e
** **	Bacteria	ee37af62729e550e74bf1ab402c6d303
** **	*Prevotellaceae_NK3B31*	afb2d76ff3f015fd627cfbe2c569e255
** **	*Prevotellaceae*	a76983a3bf38708183f845241cce869d
** **	*Bacteriodes*	a4ad693cd3dadd5f680a73e2fec1e867
** **	*Bacteriodes*	5599f7ca5067653572b626c8aa23a364
** **	*Bacteriodes*	840a763739f0fad66d7f93a222512069
** **	Enterobacterales	0e7abc22c6649b647f11fab3b27b4fbf
** **	*Enterococcus*	ddd8f8c09a18fa7364d8685cbb426a3d
** **	*Phascolarbacterium*	84928c196f82f7a75135b6b70cd08cb9
** **	*Megamonas*	2272d56d77c26103fc92d40454184a3c
** **	*Megasphaera*	37d28c8bef2c4a48da6f73a6cbbb9458
** **	*Caproiciproducens*	558ce6d49f66d4103395bc6499188464
** **	*Lachnospiraceae*	137aed16f7b5d28b9d31f26c6ea9682a
** **	*Lachnospiraceae*	3929267c8fe1994d4402da434c0cfa63
** **	*Lachnospiraceae*	ae90a23b25d7af4ddfbf59e38aa59d61

1 Amplicon sequence variants are single, statistically supported DNA sequences from high-throughput sequencing that can be inferred as present in the sample.

**Fig 7:**
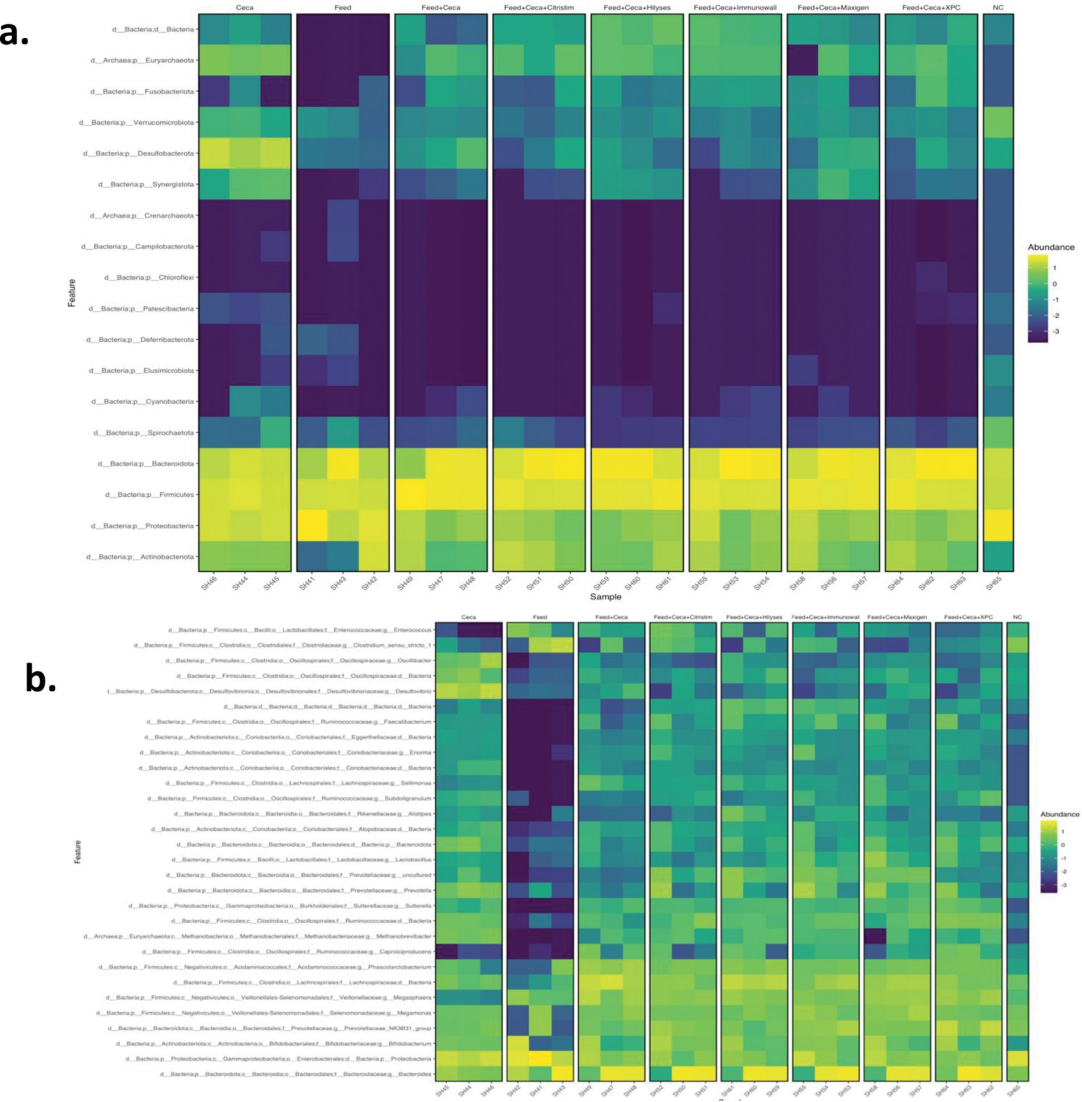
Distribution of the core microbiome members within the treatments. Distribution of the core microbiome within the five treatment groups, positive controls, and negative control for both the (**a**) phyla and (**b**) genera. Treatments include cecal contents alone, feed with no ceca or treatments, and cecal contents with feed and each respective treatment: Citristim, Hilyses, Immunowall, Maxigen, and XPC. Each treatment is represented by three samples.

To further visualize the impact of the various treatments on the prevalence of the core microbiome members, a heat map was generated (Fig 7). All five treatments demonstrated an increased abundance of the genus *Prevotella* compared to the combined FC control. The production of the SCFA, propionate, is supported by a larger abundance of *Prevotella*, which has implications for host performance, including *Salmonella* reduction, energy regulation, and immunoregulation [67–69]. *Lactobacillus*, like *Prevotella*, also produces SCFAs (61). Similar to findings in separate prebiotic studies, *Lactobacillus* was prevalent among four of the treatment groups, Hilyses, Immunowall, Maxigen, and XPC, compared to the controls [24, 68]. A higher relative abundance of *Lactobacillus* is commonly attributed to a healthy, well-balanced cecal microbiome and positive affects on growth performance [59, 70, 71]. Additionally, *Lactobacillus* has been thoroughly tested as a probiotic [72, 73]. Less is known about the effects of the growth of these cecal microorganisms on semen quality, though the limited studies have conflicting conclusions. For instance, directly introducing *Lactobacillus* into rooster semen decreases sperm movement through fermentation product acidification [74]. However, Tvrdá et al. suggest that *Lactobacillus* may improve sperm production and hatchability [75]. To definitively determine the impact of migrating cecal microorganisms into the reproductive tract, *in vivo* studies are necessary. Although increased bacterial populations may have effects on semen quality, other options, including therapeutic antibiotic use, yield another set of concerns. Tetracycline, for instance, is a therapeutic antibiotic that has historically been used in the poultry industry, though its use in rodents is known to increase abnormal sperm counts while simultaneously decreasing live sperm populations [76, 77]. Currently, there are few options for effective control of both commensal and pathogenic bacterial contamination in rooster semen, and developing a product outside of traditional antibiotics has become essential. This study offers treatments for *Salmonella* infection with the added potential of other production benefits.

## Conclusions

Our results indicate that each yeast-derived product tested can rapidly reduce *S*. Enteritidis populations by several orders of magnitude within 48 hours of *Salmonella* inoculation in rooster cecal in vitro cultures. A unique finding of this study is that Immunowall appeared to be somewhat more effective than the other yeast products, reducing *S*. Enteritidis almost -four orders of magnitude compared to the control cultures without yeast product. While these results are reflective of a rooster cecal response to various yeast-derived products, they may carry additional applications to the laying hen industry. To better understand the effects of these yeast-derived products on different aspects of the poultry industry, *in vivo* studies are necessary for both laying hens and roosters.
